# Probing the peripheral immune response in mouse models of oxaliplatin-induced peripheral neuropathy highlights their limited translatability

**DOI:** 10.12688/wellcomeopenres.16635.1

**Published:** 2021-03-29

**Authors:** Zoe Lee Hore, Sara Villa-Hernandez, Franziska Denk

**Affiliations:** 1 Wolfson Centre for Age-Related Diseases, Institute of Psychiatry, Psychology and Neuroscience, King's College London, London, SE1 1UL, UK

**Keywords:** Pain, neuropathy, chemotherapy, oxaliplatin, immune, flow cytometry, behaviour, reproducibility

## Abstract

**Background:** Chemotherapy-induced peripheral neuropathy (CIPN) is a disabling side effect of various chemotherapeutic agents, including oxaliplatin. It is highly prevalent amongst cancer patients, causing sensory abnormalities and pain. Unfortunately, as the underlying mechanisms remain poorly understood, effective therapeutics are lacking. Neuro-immune interactions have been highlighted as potential contributors to the development and maintenance of CIPN, however, whether this is the case in oxaliplatin-induced peripheral neuropathy (OIPN) is yet to be fully established.

**Methods:** In this study we used flow cytometry to examine the peripheral immune response of male C57BL/6 mice following both single and repeated oxaliplatin administration. In animals exposed to repeated dosing, we also undertook mechanical and thermal behavioural assays to investigate how oxaliplatin alters phenotype, and conducted RT-qPCR experiments on bone marrow derived macrophages in order to further inspect the effects of oxaliplatin on immune cells.

**Results:** In contrast to other reports, we failed to observe substantial changes in overall leukocyte, lymphocyte or myeloid cell numbers in dorsal root ganglia, sciatic nerves or inguinal lymph nodes. We did however note subtle, tissue-dependant alterations in several myeloid subpopulations following repeated dosing. These included a significant reduction in MHCII antigen presenting cells in the sciatic nerve and an increase in infiltrating cell types into the inguinal lymph nodes. Though repeated oxaliplatin administration had a systemic effect, we were unable to detect a pain-like behavioural phenotype in response to either cold or mechanical stimuli. Consequently, we cannot comment on whether the observed myeloid changes are associated with OIPN.

**Conclusions:** Our discussion puts these results into the wider context of the field, advocating for greater transparency in reporting, alignment in experimental design and the introduction of more clinically relevant models. Only through joint concerted effort can we hope to increase our understanding of the underlying mechanisms of CIPN, including any immune contributions.

## Introduction

Chemotherapy induced peripheral neuropathy (CIPN) is an unpleasant and debilitating side-effect of numerous neurotoxic chemotherapeutics, including epothilones, proteasome inhibitors, taxanes, vinca alkaloids and platinum-based agents ( Starobova & Vetter, 2017). Characteristically, CIPN presents itself in a ‘glove and stocking’ fashion, initially affecting the extremities before progressing proximally ( Wolf *et al.*, 2008). It typically induces a range of sensory abnormalities including paresthesias and dysesthesias, which can be exacerbated by warm or cool temperatures, impaired vibration and even sensory loss ( Staff *et al.*, 2017; Zajączkowska *et al.*, 2019). Moreover, various painful sensations have also been attributed, including burning, shooting or electric-shock-like pain ( Boland *et al.*, 2010) and increased sensitivity to both mechanical and thermal stimuli ( Flatters *et al.*, 2017; Zajączkowska *et al.*, 2019). Importantly, risk of developing CIPN is known to increase with total cumulative dose; thus it is often a dose limiting factor ( Burton *et al.*, 2007), ultimately affecting survival.

Prevalence of CIPN is markedly high, acutely affecting 60–70% of patients, with 30% continuing to suffer symptoms 6 months following cessation of chemotherapy ( Seretny *et al.*, 2014). One such agent with a particularly high incidence of inducing long-lasting peripheral neuropathy is oxaliplatin, a platinum-based chemotherapeutic commonly used in the treatment of cancers of the digestive tract, including colorectal, oesophageal, stomach, liver and pancreatic ( Zajączkowska *et al.*, 2019). Staggeringly, oxaliplatin-induced peripheral neuropathy (OIPN) has been reported to affect almost 80% of patients two years following the end of treatment ( Park *et al.*, 2011). In fact, increased incidence of numbness or tingling of the hands and feet has been noted to persist in oxaliplatin-treated patients for 6 years following cessation of chemotherapy ( Kidwell *et al.*, 2012), highlighting it as one of the more disabling agents.

Similarly, sensory deficits, primarily in the form of evoked pain-like behaviours, have been observed in various rodent models of CIPN, using a myriad of oxaliplatin-based designs ( Currie *et al.*, 2019; Gadgil *et al.*, 2019; Höke & Ray, 2014). Both acute and more chronic OIPN studies report significantly increased sensitivity to both cold ( Descoeur *et al.*, 2011; Gauchan *et al.*, 2009b; Joseph & Levine, 2009; Joseph *et al.*, 2008; Ling *et al.*, 2007b; Nassini *et al.*, 2011; Renn *et al.*, 2011) and mechanical ( Gauchan *et al.*, 2009a; Nassini *et al.*, 2011; Renn *et al.*, 2011) stimuli within the first week of exposure. Furthermore, this altered nociception often appears to outlast the treatment itself – a phenomenon comparable to clinically observed ‘coasting’, whereby neuropathy worsens or newly develops following the end of treatment ( Staff *et al.*, 2017). For example, in rodents, it has been repeatedly shown that both single ( Gauchan *et al.*, 2009a; Gauchan *et al.*, 2009b; Ling *et al.*, 2007b; Nassini *et al.*, 2011) and repeated ( Ling *et al.*, 2007a; Xiao *et al.*, 2012) administration of oxaliplatin produce behavioural deficits which persist for at least one week following the final dose. Importantly, and in line with clinical observations, similar phenotypes are also displayed in models utilising other, non-platinum based, chemotherapeutics such as paclitaxel and vincristine ( Authier *et al.*, 2003; Flatters & Bennett, 2004; Gauchan *et al.*, 2009a; Kiguchi *et al.*, 2008b; Makker *et al.*, 2017; Muthuraman *et al.*, 2008; Old *et al.*, 2014; Shen *et al.*, 2015; Xiao *et al.*, 2007), indicating the presence of common underlying mechanisms.

Despite CIPN being widely reported in both clinical and pre-clinical settings, exactly what these underlying mechanisms are is not fully understood, though development is thought to be multi-factorial ( Starobova & Vetter, 2017). Potential contributors, including dysregulation of calcium homeostasis, axon degeneration, mitochondrial dysfunction, oxidative stress and alterations to ion channels and the immune response have all been proposed ( Flatters *et al.*, 2017; Starobova & Vetter, 2017). In fact, this latter notion, which suggests a role for neuro-immune interactions, has garnered considerable attention in recent years ( Lees *et al.*, 2017), with studies demonstrating altered immune responses to various chemotherapy drugs. Centrally, multiple groups have reported an increase in spinal microglia ( Burgos *et al.*, 2012; Kiguchi *et al.*, 2008a; Peters *et al.*, 2007; Ruiz-Medina *et al.*, 2013; Shen *et al.*, 2015), though there has been some debate regarding whether microglia or astrocytes contribute to the pathogenesis of CIPN ( Robinson *et al.*, 2014; Zhang *et al.*, 2012). Peripherally, there is strong evidence supporting the involvement of immune cells. For example, repeated administration of vincristine is reported to induce macrophage infiltration into the sciatic nerves and DRG ( Kiguchi *et al.*, 2008b; Old *et al.*, 2014). Similarly, following two 18mg/kg i.v. injections of paclitaxel, increased expression of the macrophage marker CD68 has been observed in these peripheral tissues ( Peters *et al.*, 2007). In fact, even a single 6mg/kg dose of paclitaxel has been shown to result in increased incidence of leukocytes in the DRG, including macrophage, monocyte, neutrophil and T cell populations ( Liu *et al.*, 2014). Furthermore, in the case of both chemotherapy agents, disruption of the inflammatory response is associated with improvement of behavioural deficits, highlighting the importance of such immune cells in the development of painful phenotypes ( Liu *et al.*, 2014; Old *et al.*, 2014).

Although oxaliplatin is one of the most utilised drugs in CIPN models ( Currie *et al.*, 2019), its effects on the immune response have been relatively poorly investigated. Of the limited number of studies conducted, many have focused only on tissues of the central nervous system ( Cho *et al.*, 2016; Di Cesare Mannelli *et al.*, 2014; Janes *et al.*, 2015), while the handful of peripheral approaches in the literature have largely not employed flow cytometry techniques, relying on immunohistochemical analysis ( Di Cesare Mannelli *et al.*, 2013; Li *et al.*, 2016) and whole tissue qPCR ( Marmiroli *et al.*, 2017). In potentially the only peripherally focused, flow cytometry-heavy investigation of OIPN, oxaliplatin-induced increases were noted specifically in circulating T cell populations, though such differences were not detected in either the lumbar DRG or sciatic nerves ( Makker *et al.*, 2017). However, their flow cytometric investigations did not extend to myeloid cell types in the periphery, such as macrophages, which have been implicated in the development and even maintenance of neuropathic pain in a variety of models ( Kiguchi *et al.*, 2019; Ristoiu, 2013), including nerve injury ( Liang *et al.*, 2019; Yu *et al.*, 2020).

We are therefore lacking more detailed accounts regarding the specific effects of oxaliplatin on the peripheral immune response, and in particular, on innate cell types. Furthermore, the fact reports to date have been somewhat conflicting, makes interpretation of the current OIPN literature ambiguous. For example, lumbar DRG expression of the macrophage/microglial marker ionized calcium binding adaptor molecule 1 (IBA1) has been shown to both increase ( Li *et al.*, 2016) and remain unaltered ( Makker *et al.*, 2017) following repeated oxaliplatin administration. Meanwhile, another study completely failed to detect macrophages in either the L4–5 DRG or sciatic nerves following 3 weeks of oxaliplatin treatment ( Di Cesare Mannelli *et al.*, 2013).

The aim of this study is therefore to utilise immunological approaches in order to clarify whether there is peripheral dysregulation of the innate and/or adaptive immune systems in response to both single and repeated oxaliplatin administration, and if so, speculate how this may contribute to the generation and maintenance of painful peripheral neuropathies.

## Methods

### Ethical considerations

All experiments described were carried out in accordance with the United Kingdom Home Office Legislation ( Animals (Scientific Procedures) Act, 1986, 2021) and were approved by the Home Office to be carried out at King’s College London under project license number P57A189DF. This study is reported in line with the Animal Research: Reporting of In Vivo Experiments (ARRIVE) guidelines ( Hore *et al.*, 2021a).

All efforts were made to ameliorate harm to animals used in this study. This was achieved in a number of ways, including:

- Provision of housing that allowed expression of species-specific behaviours, such as ample bedding material and tunnels, and wherever possible, group housing.
- Provision of extra food and diet supplements to mice who were undergoing repeated dosing and were at risk of losing weight.
- Regularly weighing mice and keeping a close eye on their health to ensure that any animals displaying signs of illness could be assisted rapidly and not left to suffer.
- Ensuring new needles were used for each animal during dosing and rotating injection side in animals who underwent repeated dosing.
- Regular handling of animals prior to and during behavioural testing to familiarise them with being handled and minimise any distress.

### Animals

Male C57BL/6JOlaHsd mice were purchased from Envigo at 8–13 weeks of age and acclimatised to the animal unit for one week prior to any procedures. Mice that were purchased at 13 weeks were singly housed within one week of arrival due to fighting between cage mates. For all experiments, mice were housed in standard individually ventilated cages (Tecniplast) in groups of five maximum at a 12h light–dark cycle, with *ad lib* access to food and water.

### Chemotherapy induced peripheral neuropathy (CIPN) models

We conducted two separate studies, designed to measure the effect of oxaliplatin versus vehicle treatment. Mice were arbitrarily assigned to either group, in a manner that allowed for cage mate controls, and subjected to one of the following dosing regimens. In both instances, mice were returned to their home cage immediately after injection.

*Single administration (n=6 mice):* The oxaliplatin solution was made up on the day of injection by diluting 1.2mg of oxaliplatin powder (Sigma, #O9512) in 2ml of 5% glucose to give a final concentration of 0.6mg/ml. A single 6mg/kg dose of oxaliplatin, or an equal volume of 5% glucose, was administered intraperitoneally (i.p.) and mice were weighed prior to, and for 2 days following injection.

*Repeated administration (n=10 mice):* The oxaliplatin solution was made up at the beginning of each dosing week by diluting the stock oxaliplatin (5mg/ml) (Guy’s Cancer Centre, Accord) 1:10 with 5% glucose. Mice were administered a single 3mg/kg dose of oxaliplatin or an equal volume of 5% glucose, daily for 5 days – this constituted 1 cycle of injections. This was repeated for 4 cycles, with mice given a dosing break of 1 week after the first 2 cycles. Note that on the final week of dosing mice were only injected 4 times (Monday-Thursday), as 2 mice (1 oxaliplatin, 1 vehicle) were found dead in their cages shortly after dosing on Thursday morning. A single mouse from the oxaliplatin group was also found dead 2 days prior to this. Mice were weighed daily during injection cycles and every other day during the dosing break week. All mice were supplemented with DietGel® (ClearH _2_O, #72–07–5022) or monkey nuts 2–3 times per week, regardless of whether they had lost weight or not.

Sample sizes used in the single administration study were determined based on the number of animals commonly used in published studies of a similar nature. Sample sizes were increased in the repeated administration study to allow for larger *n* numbers in behavioural assays, in which inter-animal variability is commonly observed, and to account for potential loss of animals due to the much higher cumulative dose of oxaliplatin animals were receiving. Note that the only set criteria for exclusion of animals from experimental procedures was if they were noticeably unwell. Humane endpoints set out for this work included exceeding a moderate level of pain, defined either by excessive weight loss or signs of pain that extend beyond neuropathy (e.g. hunched posture, ungroomed coat). However, no animals displayed overt signs of illness during the study or exceeded any endpoint limits, thus no animals were excluded from experiments or prematurely culled.

### Behavioural assays

All behavioural tests were conducted in adult mice (14 weeks or older), within mouse behavioural testing rooms at King’s College London. Baseline behavioural testing took place the week prior to mice receiving their first injection, while all further testing was carried out between 24 hours and 36 days of mice receiving their first dose. Note that behavioural assays performed on day 36 were undertaken 6 days following the last injection. For a given time point, both tests were performed on the same day. Two different individuals carried out the oxaliplatin dosing and the behavioural assays, thus the experimenter was blind to treatment group throughout testing and until all behavioural data had been analysed.

*von Frey:* Mice were acclimatised to the testing arena - a Perspex chamber on a wire mesh floor - for 1 hour on one day prior to the start of testing. On the 11 testing days, mice were arbitrarily assigned to chambers and left to acclimatise for at least 1 hour. Withdrawal thresholds were determined using a simplified version of the up and down method ( Bonin *et al.*, 2014) with a range of von Frey filaments (0.04–2g) (Touch Test, North Coast Medical, Inc.). Briefly, calibrated filaments were applied to the plantar surface of each hind paw, at a force strong enough for the filament to bend slightly, for 3 seconds or until the animal withdrew its paw. A 50% paw-withdrawal threshold was calculated as previously described ( Bonin *et al.*, 2014). For baseline readings, two tests were conducted, and a 50% paw withdrawal threshold was calculated as an average of the two.

*Cold plate:* Mice were acclimatised to the testing arena – a switched-off 20cm diameter incremental hot/cold plate surrounded by a transparent acrylic cylinder (Ugo Basile) – for five minutes on one day prior to testing. On the 7 testing days, animals were acclimatised to a switched-off plate for 2 minutes before being transferred to an identical cold plate set at 10°C. Mice were observed for a response (jumping, hind paw shaking or hind paw licking) and their latency to respond was recorded. If a mouse made a jump response it was immediately removed from the arena and returned to its home cage. A maximum latency of 90 seconds was set to prevent damage to the plantar skin. To ensure that the correct latencies were noted, each time point was recorded, and the videos were re-scored. If the live and re-scored latencies differed, the re-scored time was taken. Any mice that failed to respond were awarded the maximum latency of 90 seconds.

### Tissue processing

In accordance with the approved methods of euthanasia set out in the licence we were working under, either 4 days (single dose model) or 38–39 days (repeated dosing model) following their first injection, mice were deeply anaesthetised via overdose of pentobarbital (Euthatal; Merial, Lot# P02601A) administered i.p.. Once unresponsive, animals were perfused with 10ml of 1x PBS to avoid blood contamination. Following sacrifice, a laminectomy was performed in order to expose the lumbar spinal cord. To ensure the correct DRG were taken, the sciatic nerves were exposed and followed up towards the spinal cord to identify and dissect out L3-L5 DRG into F12 (Gibco, # 21765-029). The sciatic nerves themselves were then dissected out into a petri dish containing F12 and cut to 0.5cm. Lastly, the right and left inguinal lymph nodes were exposed and dissected out into F12, trying to separate them as much as possible from the surrounding fat. Tissues were kept in F12 on ice until all animals in a given batch were processed, they were then dissociated largely in accordance with previously described methods ( Liang *et al.*, 2020). Briefly, tissues were transferred into 50µl of digestion mix and incubated at 37°C, shaking at 220RPM for 45 minutes (see Table 1 and Table 2 for digestion mixes used for each tissue type). To achieve optimal digestion, nerves and inguinal lymph nodes were chopped into small pieces with spring scissors (50 and 30 chops, respectively) prior to incubation. Following digestion, samples were centrifuged, supernatants removed, and the remaining pellets were resuspended in 100μl of FACS buffer (see Table 3 for composition). Samples underwent dissociation via repeated up-down pipetting using a P200 (30x for DRG and inguinal lymph nodes; 50x for sciatic nerves) and were then filtered through the 35μm cap of a BD Falcon 12 × 75 mm tube with cell strainer cap (BD Biosciences, # 352235) into a 96 well v-bottom plate (Thermo Scientific, # 612V96). Finally, the plate was centrifuged, supernatants discarded and the remaining pellets underwent antibody staining as described in the following.

**Table 1.** Digestion mix used in processing of L3-5 dorsal root ganglia (DRG) samples.

Reagent (supplier, cat #)	Final concentration
F12 (Gibco, 21765-029)	-
Dispase ll (Sigma Aldrich, 04942078001)	3mg/ml
Collagenase type IA (Sigma Aldrich, C9891)	12.5mg/ml
DNase l (Sigma Aldrich, 10104159001)	10mg/ml

**Table 2.** Digestion mix used in processing of sciatic nerve and inguinal lymph node samples.

Reagent (supplier, cat #)	Final concentration
F12 (Gibco, 21765-029)	-
Collagenase type IA (Sigma Aldrich, C9891)	6.25mg/ml
Pronase (Millipore, 53702)	0.2%
Hyaluronidase (ABNOVA, P52330)	0.4%

**Table 3.** Fluorescence-activated cell sorting (FACS) buffer composition.

Reagent (supplier, cat #)	Final concentration
HBSS (Gibco, 14175095)	-
BSA (Sigma-Aldrich, A3983)	0.4%
HEPES (Gibco, 15630080)	15mM
EDTA (Invitrogen, 15575038)	2mM

### Flow cytometry

Staining of samples for flow cytometry was conducted as described previously ( Liang *et al.*, 2020). Briefly, to distinguish live cells, samples were incubated in a fixable yellow viability dye (Invitrogen, # L34959) for 30 minutes, followed by 30 minutes incubation in a mix of directly conjugated antibodies and Fc block (see Table 4 for antibody panel and concentrations). Following centrifugation, the staining mix was removed and remaining pellets were incubated for 5 minutes in 4% paraformaldehyde (PFA) for fixation. Once fixed, samples were centrifuged, PFA removed and pellets resuspended in 200μl of FACS buffer. Flow cytometry was conducted on a BD Fortessa at the NIHR BRC flow core facility at King’s College London, with compensation controls employed as described previously. All analysis was carried out using FlowJo version 10.6.0 software (see Extended Figure 1 for gating strategies employed ( Hore *et al.*, 2021a)).

**Table 4.** Previously optimised ( Liang *et al*., 2020) antibody panel used for all flow cytometry experiments.

Laser	Colour	Epitope	Cell Type	Final Dilution	Mono/ polyclonal	Species	Cat #
UV	BUV395	Ly6G	Neutrophils	1:300	Monoclonal	Rat anti-mouse	BD Bioscience, #563978
Violet	AmCyan	Live/ Dead	-	1:1000	N/A	N/A	Invitrogen, #L34959
	BV650	Ly6C	Monocytes	1:1500	Monoclonal	Rat anti-mouse	BioLegend, #128049
Blue	FITC	CD45	Leukocytes	1:1200	Monoclonal	Rat anti-mouse	BioLegend, #103108
Yellow	PE-Cy7	CD11b	Myeloid lineage	1:1200	Monoclonal	Rat anti-mouse, human	BioLegend, #101215
	PE	β -TCR	T cells (αβ chain)	1:300	Monoclonal	Armenian hamster anti-mouse	BioLegend, #109207
Red	APC-Cy7	MHCII	Activated macrophages & dendritic cells	1:1200	Monoclonal	Rat anti-mouse	BioLegend, #107628
	APC	δ-TCR	T cells (γδ chain)	1:300	Monoclonal	Armenian hamster anti-mouse	BioLegend, #118116
-	-	Fc block	CD16/32	1:20	Monoclonal	Rat anti-mouse	BioLegend, #101302

### Harvesting and culture of bone marrow derived macrophages (BMDMs)

Harvesting and culture of BMDMs was conducted as described previously ( Liang *et al.*, 2020). Briefly, femur and tibia bones from both hind limbs were collected and the bone marrow was flushed out with cold PBS. Following centrifugation and filtration, the cell suspension was plated onto 15cm Petri dishes and incubated for 5–7 days to allow for differentiation into mature naïve macrophages. After 5–7 days of incubation, cells were gently dislodged with a cell scraper (Greiner, #541–070) and incubated for a further 24hrs in DMEM (Gibco, #32430–027) + MCSF (PeproTech, #315–02). The following day, cells were incubated for 4hrs in 10ng/ml of TNFα (BioLegend, #575202), while unstimulated cells were incubated in plain DMEM as a control. For RNA extraction, we used an RNAeasy Microkit 50 (Qiagen, #74004) following manufacturer’s instructions. mRNA quantity was evaluated using a Qubit 3 Fluorometer (Invitrogen).

### Reverse transcription quantitative real-time polymerase chain reaction (RT-qPCR) of BMDMs

1ng of the extracted RNA was used to synthesize cDNA using the Smart-Seq2 protocol, 16 cycles of amplification ( Picelli *et al.*, 2014). Where appropriate, the resultant cDNA was diluted down to 1ng/µl with double distilled H _2_O (ddH _2_O). 1ng of cDNA was used in standard SYBR Green RT-qPCR reactions, whereby 1µl of cDNA was added to a mix comprised of 5µl LightCycler® 480 SYBR Green I Master (Roche, #42352720), 1µl of relevant primer mix (10µM) and 3µl of ddH _2_O. Samples were run on a LightCycler® 480 Instrument II (Roche, #05015243001) to probe for genes of interest (see Table 5 for primer sequences). All primers were tested for their efficiency and specificity prior to use. The housekeeping gene GAPDH was used to calculate ΔΔCt values. All reactions were run in duplicate with water used as a negative control.

**Table 5.** List of primers used to probe for genes associated with DNA damage, apoptosis and cellular stress.

Gene	Function	Primer sequence
GAPDH forward	Housekeeping	GGCCTTCCGTGTTCCTAC
GAPDH Reverse		TGTCATCATACTTGGCAGGTT
TRP53 forward	Cell cycle regulator, apoptosis inducible	GTCACAGCACATGACGGAGG
TRP53 reverse		TCTTCCAGATGCTCGGGATAC
GADD45A forward	DNA damage/stress	CCGAAAGGATGGACACGGTG
GADD45A Reverse		TTATCGGGGTCTACGTTGAGC
PUMA forward	Pro-apoptotic gene	GCGGCGGAGACAAGAAGA
PUMA reverse		AGTCCCATGAAGAGATTGTACATGAC
FOS forward	Cell stress transcription factor	CGGGTTTCAACGCCGACTA
FOS reverse		TTGGCACTAGAGACGGACAGA
SESN2 forward	Cell growth and survival regulator	TCCGAGTGCCATTCCGAGAT
SESN2 reverse		TCCGGGTGTAGACCCATCAC
DRAM1 forward	DNA damage/autophagy	TCATCTCCTACGTGGTCGC
DRAM1 reverse		CTGCGCCAAGAAATGCAGAG
MDM2 forward	P53 regulator	TGTCTGTGTCTACCGAGGGTG
MDM2 reverse		TCCAACGGACTTTAACAACTTCA
PTEN forward	Cell growth regulator	TGGATTCGACTTAGACTTGACCT
PTEN reverse		GCGGTGTCATAATGTCTCTCAG
F4/80 forward	Myeloid cells/macrophages	TGACTCACCTTGTGGTCCTAA
F4/80 reverse		CTTCCCAGAATCCAGTCTTTCC

### Outcome measures

In this study, the following outcome measures were assessed, and comparisons were made between oxaliplatin and vehicle treated mice.

*Behavioural assays*: Cold plate = latency to jump or latency of hind paw response, namely shaking or licking (seconds), von Frey = 50% hind paw withdrawal threshold (grams).

*Flow cytometry experiments*: Total number of live single cells per population of interest.

*BMDM experiments*: Total macrophage cell number, based on haemocytometer counts under a light microscope. Expression of genes associated with DNA damage, apoptosis and cellular stress, based on ΔΔCt values calculated from data generated in RT-qPCR experiments.

### Statistical analysis

For analysis of the flow cytometry data, comparisons between the oxaliplatin and vehicle groups were carried out using either an unpaired t-test or Mann-Whitney test, depending on normality (Shapiro-Wilk test). Data from two animals were excluded, or partially excluded from analysis - in accordance with the ARRIVE 2.0 reporting guidelines ( du Sert *et al.*, 2020), details of these exclusions can be found in Extended Table 1 ( Hore *et al.*, 2021a). For behavioural tests and weights, repeated measures two-way ANOVAs were conducted, followed by Sidak’s multiple comparisons tests to assess differences between the two groups at a given timepoint and between timepoints within each group. For BMDM experiments, paired and multiple t-tests were used. In all cases significance was set at *p*=<0.05. All statistics were performed using GraphPad Prism version 9.0.0 software.

Effect sizes mentioned in the discussion and Extended Table 2 ( Hore *et al.*, 2021a) were obtained by calculating Cohen’s *d* using the equation *(Mean _2_-Mean _1_)/√((SD _1_^2^+SD _2_^2^)/2)*. We calculated power functions for non-parametric and parametric two-tailed t-tests, and for repeated measures ANOVAs (between factors) for a range of different effect and sample sizes, to help assess the likely sensitivity of our flow cytometry experiments and published behavioural tests (Extended Figure 4 and Figure 5 ( Hore *et al.*, 2021a)). For this we used G*Power version 3.1.9.7 software with the following parameters – t tests (Means: Difference between two independent means), y axis: power (1-β err prob), as a function of: Effect size *d* (from 0 through to 2), α err prob: 0.05, for a range of sample sizes from *n*=6 to *n*=16 or *n*=4 to *n*=18. F tests (ANOVA: Repeated measures, between factors), y axis: power (1-β err prob), as a function of: Effect size *f* (from 0 through to 1), α err prob: 0.05, for a range of sample sizes from *n*=6 to *n*=16.

### Open-access software alternatives

*GraphPad Prism:* R is a language and environment for statistical computing which can be used to conduct all statistical analysis carried out in this study and create accompanying graphs.

*FlowJo:* All flow cytometry analysis can be conducted using Flowing Software 2.5.1, which is available to download for free.

## Results

### A single dose of 6mg/kg oxaliplatin resulted in short-term weight loss which resolved within 2-days following injection

**Figure 1.**
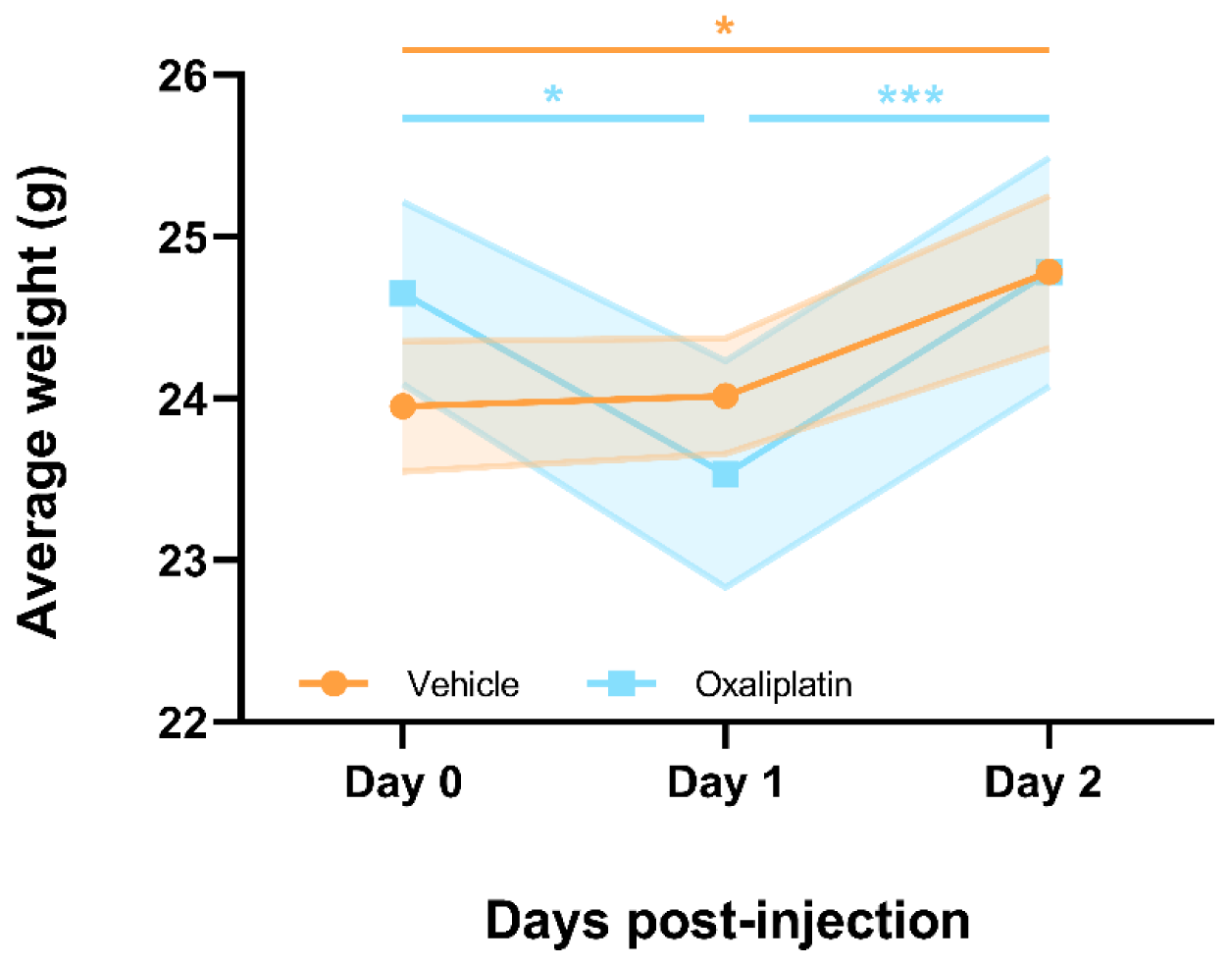
Mice receiving a single 6mg/kg dose of oxaliplatin lose weight acutely but appear to recover within 48hrs of drug administration. Oxaliplatin treated mice lost a significant amount of weight 1 day following drug administration, however, by day 2 mice had already returned to their pre-injection weights. Meanwhile, vehicle treated mice gained a significant amount of weight over the 2 days following injection. Data displayed as mean ±SEM, ( *n*=6). RM Two-way ANOVA with Sidak’s multiple comparisons test revealed a significant main effect of time (F(1.683, 16.83)=29.08, p<0.0001) and interaction between time and group (F(2,20)=10.12, p=0.0009). * p<0.05, *** p<0.001.

In order to gain an indication of whether oxaliplatin was having a negative effect on the health of these mice - suggesting effective administration - the body weights of all animals were monitored prior to injection i.e. day 0, and for 2 days following injection i.e. day 1 and 2 ( Figure 1 ( Hore *et al.*, 2021a)). The oxaliplatin group lost a significant amount of weight within 1 day of injection (day 0 - day 1: *p*=0.0170), however, mice had, on average, returned to their pre-injection weights by the second day following dosing. On the other hand, their vehicle treated counterparts steadily gained weight following injection (day 0 – day 2: *p*=0.0129). These data provide some indirect evidence for oxaliplatin having been successfully administered. However, they also suggest that a single dose of oxaliplatin negatively affects the health of a mouse for only a short period of time after administration. No behavioural assays were conducted for this acute administration model, and flow cytometry was performed 4-days following injection i.e. once animals had returned to their pre-injection weights.

### Acute oxaliplatin administration did not alter total leukocyte number in lumbar DRG, sciatic nerves or inguinal lymph nodes

**Figure 2.**
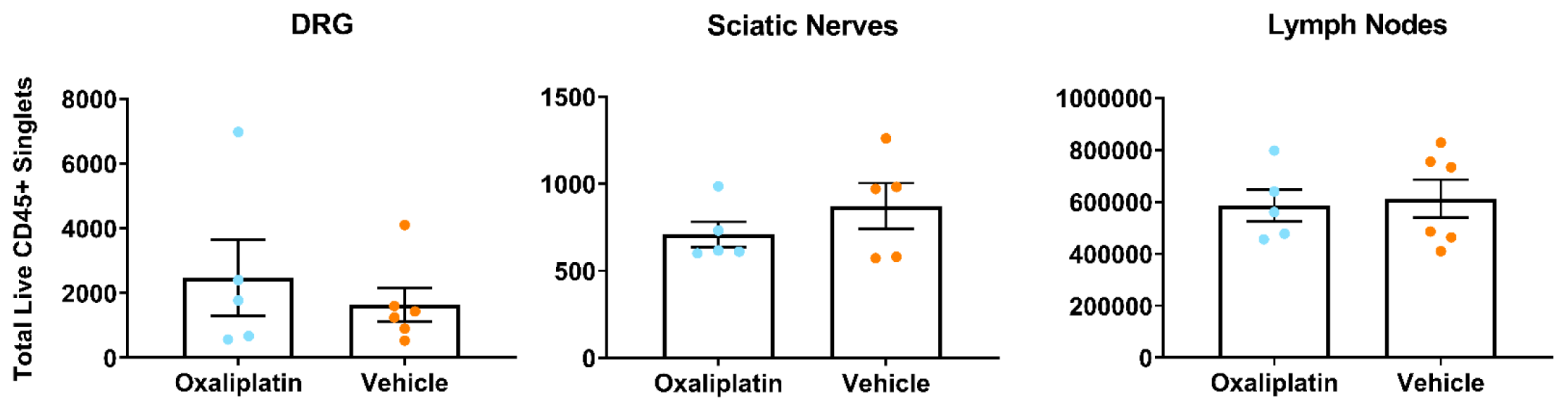
Total leukocyte numbers did not differ between vehicle and oxaliplatin treated mice in any tissue type following acute administration of oxaliplatin. Analysis of the total number of CD45+ live single cells from flow cytometry of lumbar 3–5 dorsal root ganglia, sciatic nerves and inguinal lymph nodes. Data displayed as individual animals ±SEM, ( *n*=5-6). Unpaired t-test or Mann-Whitney test, depending on whether the data were normally distributed (Shapiro-Wilk test).

Based on reports from the literature that even acute oxaliplatin-based CIPN models induce prolonged behavioural deficits ( Descoeur *et al.*, 2011; Gauchan *et al.*, 2009a; Gauchan *et al.*, 2009b; Joseph *et al.*, 2008; Ling *et al.*, 2007b; Nassini *et al.*, 2011), we hypothesised that if neuro-immune interactions are implicated in this phenomenon, any associated changes in peripheral immune profile should be observed in such a model. All samples were processed in a single batch, 4 days following a single i.p. injection of oxaliplatin (6mg/kg) or an equal volume of vehicle, and run together on a flow cytometer, using a previously optimised panel (see Table 4 and Extended Figure 1 ( Hore *et al.*, 2021a ) for panel and gating strategies employed). Contrary to our expectations, analysis of our dataset did not reveal any differences in total leukocyte number (CD45+ cells) between oxaliplatin and vehicle groups for any tissue type ( Figure 2 ( Hore *et al.*, 2021c)). This was largely reflected in both the lymphoid ( Extended Figure 2 ( Hore *et al.*, 2021a)) and myeloid subpopulations we looked at, with the exception of MHCII+ cells, which were significantly downregulated in the lymph nodes of oxaliplatin treated animals ( *p*=0.0230) ( Extended Figure 3 ( Hore *et al.*, 2021a)).

### Repeated oxaliplatin administration resulted in failure to gain weight

**Figure 3.**
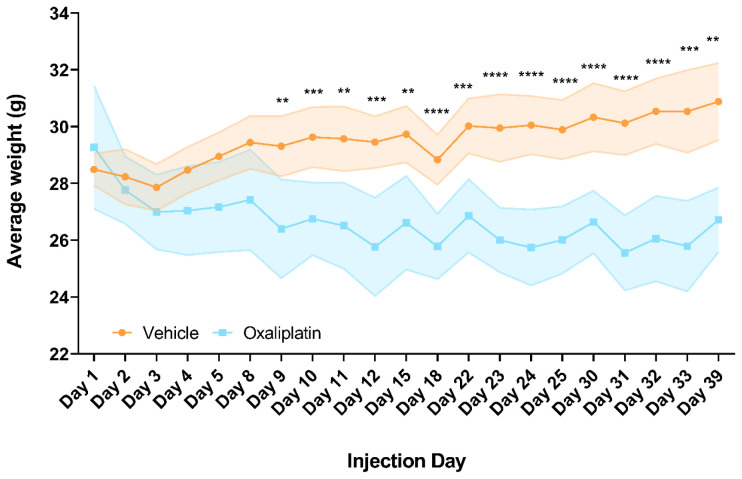
Repeated oxaliplatin treatment significantly impacted weight gain. Oxaliplatin treated mice started to lose weight within 1 day of dosing and became significantly lighter than the vehicle group by day 9. This trend continued throughout dosing until 6 days following the last injection i.e. ‘Day 39’, where oxaliplatin mice on average weighed 4.3 grams less than their vehicle treated counterparts. Note that days 15 and 18 were time points during the non-dosing week. Mice used for flow cytometry batches 1 and 2 were collected on days 38 and 39, respectively. Data displayed as mean ±SD, (days 1-30: *n*=10, days 31-32: *n*=9-10, day 33: *n*=8-9, day 39: *n*=5-6). RM Two-way ANOVA with Sidak’s multiple comparisons test revealed a significant main effect of group (F (1, 18) = 35.11, p=<0.0001) and interaction with time (F (20, 346) = 22.66, p=<0.0001). ** p<0.01, *** p<0.001, **** p<0.0001.

As we largely failed to detect alterations in the peripheral immune response at any level following acute administration of oxaliplatin, we proceeded to investigate if we could detect changes using a paradigm with more continuous drug delivery. We used 4 dosing cycles over a period of a month (19 injections in total). Such a model better reflects the clinical situation, where patients on average undergo 4 to 8 cycles of treatment over a period of 3–6 months (Cancer Research UK, 2018). We monitored the weight of all mice throughout treatment and observed a reduction in the weights of oxaliplatin treated mice within 24 hours of the first injection, a trend which persisted until the end of the experiment. Although the oxaliplatin group was not significantly lighter comparing the start and end of the experiment ( *p*=0.5122), they were significantly lighter than their vehicle treated counterparts by day 9, i.e. the 7 ^th^ treatment day ( *p*=0.0087) and remained so until the end of the experiment ( *p*=0.0047). No recovery of weight loss was observed in the treatment break week (days 15 and 18). Meanwhile, vehicle treated mice gradually gained weight over the testing period, though were not significantly heavier by the end of the experiment ( *p*=0.1093) ( Figure 3). Although we did not perform tissue specific assays to assess platinum concentrations, as other studies have done ( Canta *et al.*, 2011; Marmiroli *et al.*, 2017), these results strongly indicate that oxaliplatin was having a systemic effect and therefore is likely to have reached the tissues of interest in this study i.e. sciatic nerves, lumbar DRG and inguinal lymph nodes.

### Repeated oxaliplatin administration did not alter total leukocyte number in lumbar DRG, sciatic nerves or inguinal lymph nodes

**Figure 4.**
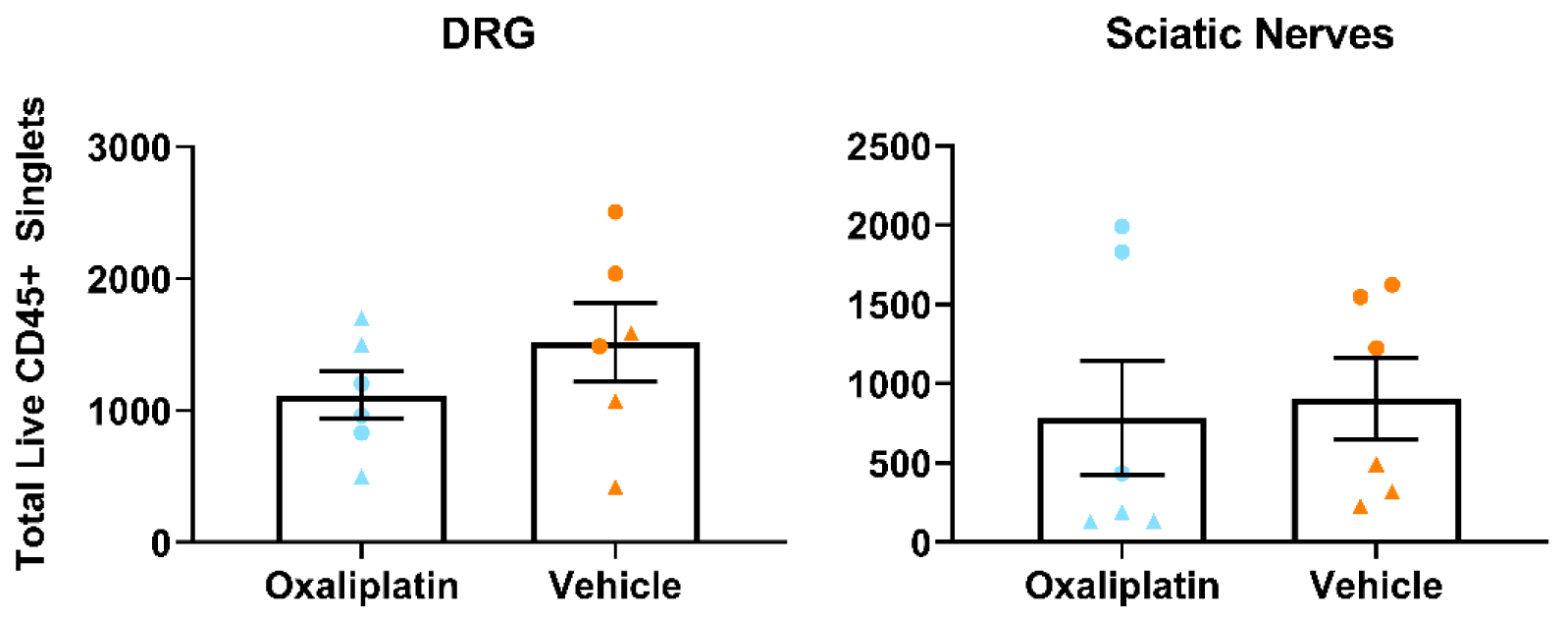
Total leukocyte numbers did not differ between vehicle and oxaliplatin treated mice in any tissue type following repeated administration of oxaliplatin. Analysis of total number of CD45+ live single cells from flow cytometry of lumbar 3-5 dorsal root ganglia, sciatic nerves and inguinal lymph nodes. Points of the same shape within each group were processed in the same batch. Data displayed as individual animals ±SEM, ( *n*=6). Unpaired t-test or Mann-Whitney U test, depending on whether the data were normally distributed (Shapiro-Wilk test).

For this experiment, mice were processed in two batches (n=3/day) 38–39 days following their first injection of oxaliplatin (3mg/kg), or equal volume of vehicle, and all samples were run together on a flow cytometer (see Table 4 and Extended Figure 1 ( Hore *et al.*, 2021a) for panel and gating strategies employed). As was the case with the acute model, our data failed to show any differences in total leukocyte number (CD45+ cells) between oxaliplatin and vehicle groups ( Figure 4 ( Hore *et al.*, 2021b)). Average cell numbers were comparable to those obtained from mice who had only received a single dose of oxaliplatin ( Figure 2).

We observed substantial inter-animal variability and therefore considered whether we might be able to detect a relationship between cell numbers and processing batch or drug treatment group. We were particularly interested in the latter question, given that chronic OIPN is evident in only about 70% of patients ( Molassiotis *et al.*, 2019; Park *et al.*, 2013). However, we did not detect any convincing correlations with either of these variables. Simple examination of the data ( Figure 4) already indicates that there is no clear group difference in variability, and while some batch-associated variability was present, this was restricted to the sciatic nerve. To be sure, we also correlated total leukocyte number with behavioural data from each animal to see if there was a relationship between an animal’s sensitivity to mechanical and cold stimuli and its immune profile, but we were unable to detect any such trend (data not shown).

### Repeated oxaliplatin administration caused subtle changes to a subset of myeloid lineage subpopulations

**Figure 5.**
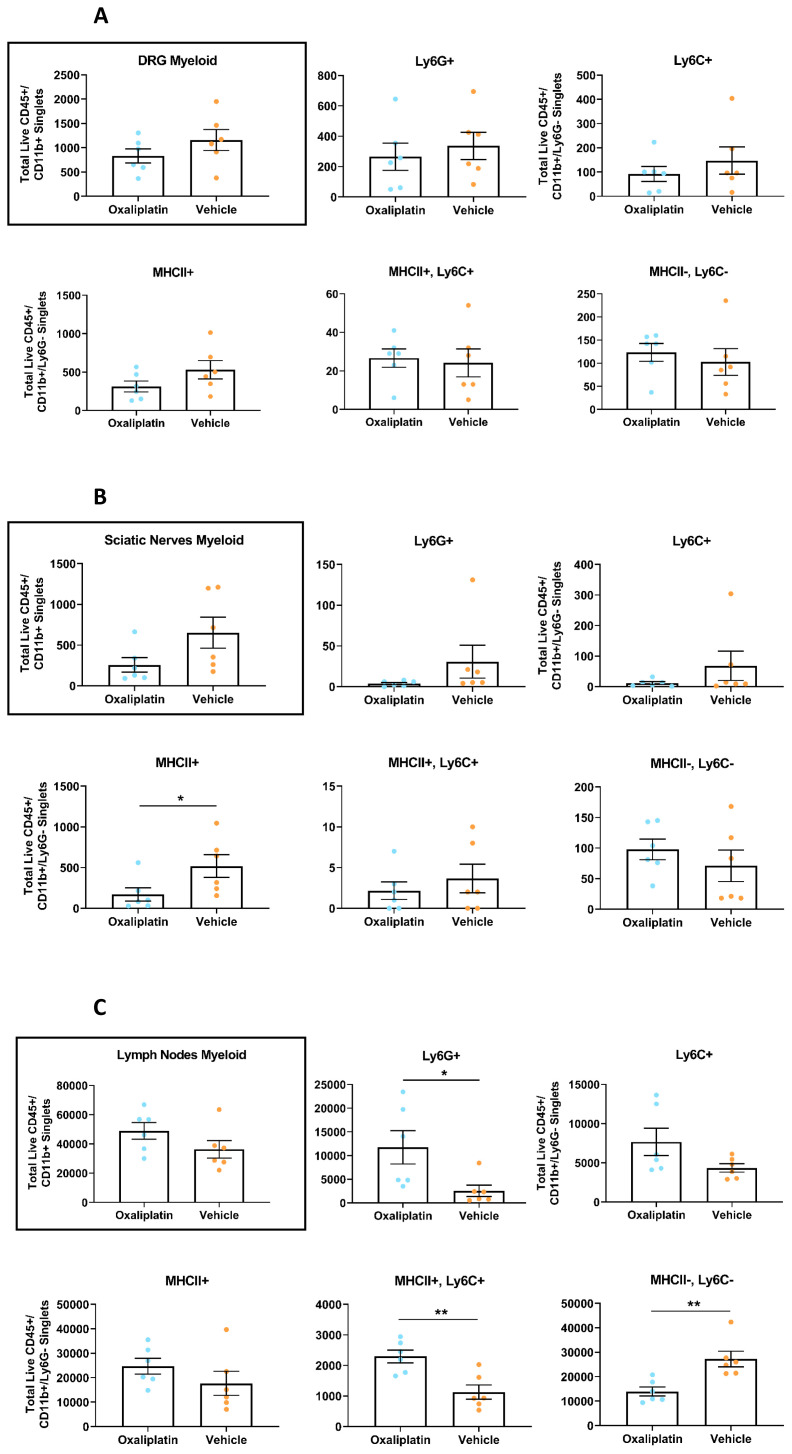
Following repeated oxaliplatin treatment total myeloid cell numbers did not differ between groups in any tissue type, however some myeloid subpopulations were dysregulated in a tissue dependant manner. Flow cytometry revealed no differences in total number of myeloid cells (CD45+/CD11b+) or infiltrating monocytes (CD45+/CD11b+/Ly6G-/MHCII-, Ly6C+) between groups for any tissue type. However, repeated oxaliplatin treatment appeared to reduce the number of MHCII antigen-presenting cells (CD45+/CD11b+/Ly6G-/MHCII+, Ly6C-) in the sciatic nerves and resident macrophages (CD45+/CD11b+/Ly6G-/MHCII-, Ly6C-) in the inguinal lymph nodes. Furthermore, in the inguinal lymph nodes both the double positive population (CD45+/CD11b+/Ly6G-/MHCII+, Ly6C+), which are likely infiltrating monocytes differentiating into resident populations, and the neutrophil population (CD45+/CD11b+/Ly6G+) were significantly upregulated in the oxaliplatin group. Analysis of flow cytometry results from ( **A**) Lumbar 3-5 dorsal root ganglia; ( **B**) Sciatic nerves; ( **C**) Inguinal lymph nodes. Data displayed as individual animals ±SEM ( *n*=6). Unpaired t-test or Mann-Whitney U test, depending on whether the data were normally distributed (Shapiro-Wilk test), * p<0.05, ** p<0.01.

Despite observing no major immune disruption, our data did show subtle alterations in a subset of the myeloid lineage cell types we investigated, though effects were tissue dependant ( Figure 5). Within the sciatic nerves and DRG, we noted a reduction in the overall number of CD11b+ myeloid cells in oxaliplatin treated mice. In the sciatic nerve specifically, a decrease in all myeloid cell subpopulations was noted, with exception of the MHCII-/Ly6C- double negative population, in which the opposite was observed ( Figure 5B). However, none of these findings reached statistical significance, with the exception of the MHCII+ population in nerve ( *p*= 0.0260). Trends for all myeloid subpopulations were comparable between the sciatic nerve and DRG, though were consistently more exaggerated in the sciatic nerve.

Meanwhile, an inverse picture was observed for the inguinal lymph nodes ( Figure 5C). In this tissue type, an increase in total myeloid cells was observed for the oxaliplatin group, albeit statistically non-significant. Similarly, cell number directionality for individual subpopulations was in direct contrast to the DRG and sciatic nerves, with increases observed in oxaliplatin treated mice for the Ly6C+, MHCII+, MHCII+/Ly6C+ and Ly6G+ subpopulations, though this only reached significance for the latter two ( *p*=0.0041 and *p*=0.0152, respectively). Meanwhile, a significant decrease was noted in MHCII-/Ly6C- cell numbers ( *p*=0.0022).

Finally, no differences between oxaliplatin and vehicle groups were observed for lymphoid lineage cell types in any tissue ( Extended Figure 2 ( Hore *et al.*, 2021a)).

Comparing single versus repeated administration of oxaliplatin, we observed clear similarities in results between the two, with trends in both myeloid and lymphoid subpopulations consistent between paradigms in the sciatic nerve and DRG ( Figure 5, Extended Figures 2 and 3 ( Hore *et al.*, 2021a)). These similarities, however, were not evident in the lymph nodes ( Figure 5, Extended Figures 2 and 3 ( Hore *et al.*, 2021a)). For instance, acute oxaliplatin administration significantly downregulated the number of MHCII+ cells in oxaliplatin treated mice ( *p*=0.0230, Extended Figure 3C ( Hore *et al.*, 2021a)), while there was no significant change and, if anything, an increase in this cell-type in lymph nodes after repeated administration ( Figure 5C).

### Repeated oxaliplatin administration did not result in altered sensitivity to either mechanical or cold stimuli

**Figure 6.**
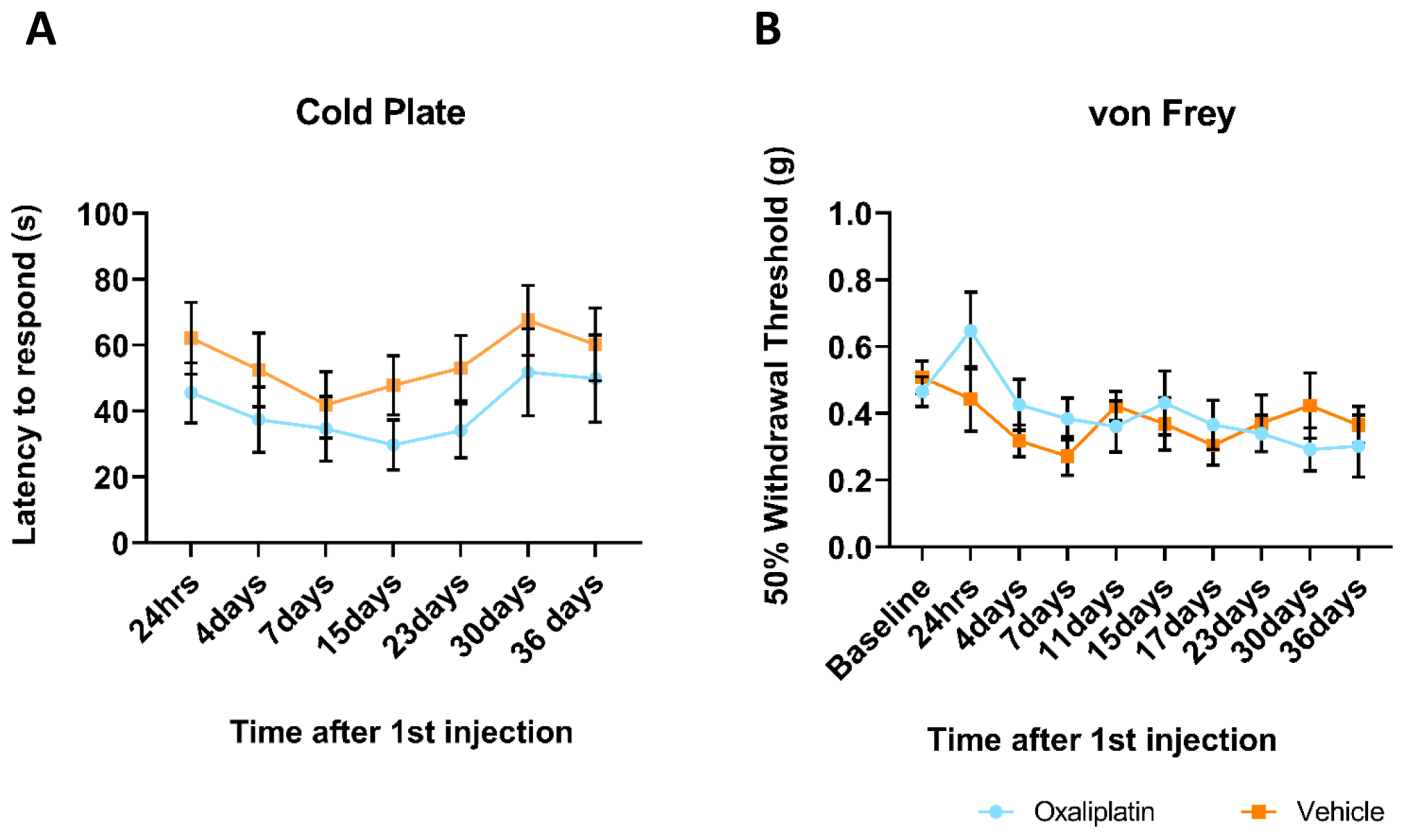
Behavioural assays revealed no difference in sensitivity to either cold or mechanical stimuli between vehicle and oxaliplatin treated mice at any time point 1-36 days following first injection. ( **A**) Although oxaliplatin treated mice had a consistently lower threshold to a 10°C cold stimuli, this did not differ significantly from the vehicle group at any time point. ( **B**) Mechanical threshold, as assessed using the von Frey test, decreased slightly over time, but did not differ between groups at any time point. Data displayed as mean ±SEM, (24hrs-23days: *n*=10, 30-36days: *n*=8-9). RM two-way ANOVA reveals no significant main effect of group (cold plate: (F (1, 18) = 1.401, p=0.2519), von Frey: (F (1, 18) = 0.1151, p=0.7384)) nor an interaction with time (cold plate: (F (6, 102) = 0.2796, p=0.9454), von Frey: (F (9, 156) = 1.330, p=0.0543)).

The fact we were unable to detect stark differences in the peripheral immune response was mirrored in the results of behavioural assays conducted on mice which underwent repeated oxaliplatin administration. Mice were tested for responses to both cold and mechanical stimuli at multiple timepoints from 1–36 days following their first injection. Results from the 10°C cold plate assay, assessed by latency to respond (hind paw shake, hind paw lick or jump), indicated that oxaliplatin treated mice did not display altered sensitivity to cold stimuli ( Figure 6A). Although the oxaliplatin group consistently showed reduced latencies throughout the testing paradigm, at no point did this differ significantly to the vehicle group. Furthermore, both groups displayed similar trajectories over time, with an initial slight decrease in response latency which had resolved by the end of the experiment (day 36). Similarly, using the up-down von Frey method ( Figure 6B), we were unable to detect differences in sensitivity to mechanical stimuli between oxaliplatin and vehicle treated mice. Both groups displayed a slight reduction in 50% withdrawal threshold over the first week of testing, which levelled out at around 0.4g from 11 days following first injection until the end of testing. For both behavioural tests, this absence of an oxaliplatin-effect was observed regardless of whether behaviour was conducted on dosing days, during the week-long dosing break (days 15 and 17) or after dosing had ceased (day 36).

### Bone marrow derived macrophages (BMDMs) harvested from mice receiving repeated oxaliplatin administration did not differ from controls

**Figure 7.**
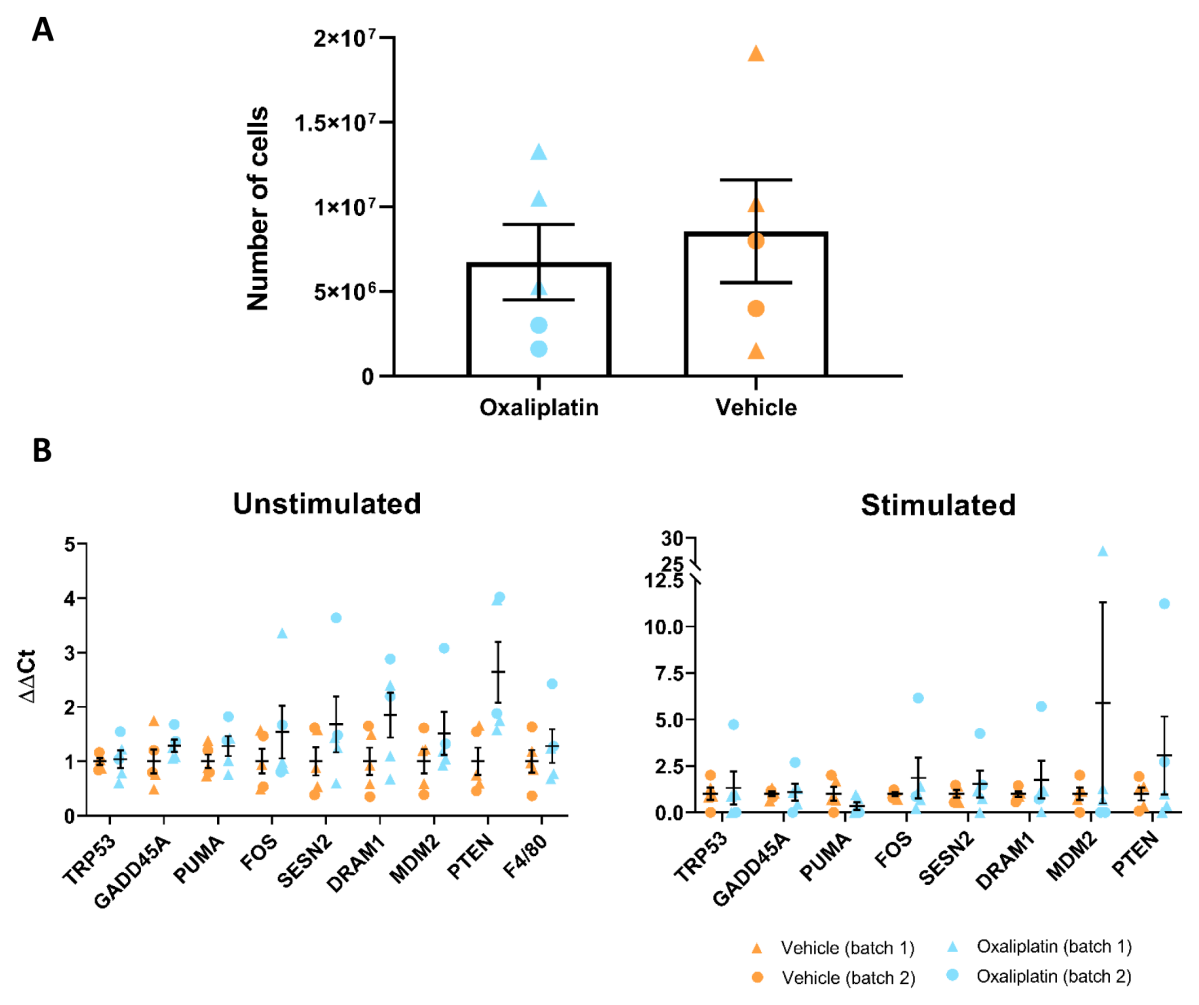
Repeated oxaliplatin administration does not alter number or phenotype of bone marrow derived macrophages (BMDMs). ( **A**) No significant difference in the overall number of cells between oxaliplatin and vehicle groups (p=0.6419). Note that these counts were taken prior to the stimulation step, where a subset of cells were incubated with TNFα. Paired t-test. ( **B**) No significant differences were observed between oxaliplatin and vehicle treated groups for any of the genes investigated. This was true for both unstimulated and stimulated BMDMs (all genes p=>0.12 and p=>0.16, respectively). Multiple t-tests. Data displayed as individual animals ±SEM, ( *n*=5).

In addition to behavioural and flow cytometry experiments, we also conducted *in vitro* work on mouse derived BMDMs, in order to see whether cell number and phenotype were altered by repeated oxaliplatin treatment. BMDMs from batch 1 mice ( *n*=3) were harvested 38 days following their first injection, meanwhile, batch 2 mice ( *n*=2) were processed 2 weeks later, thus harvesting of BMDMs from these animals took place 52 days after first injection. Prior to stimulation, cells were resuspended in PBS and counted on a haemocytometer under a light microscope. When all samples were taken together ( *n*=5), results indicated that overall macrophage numbers did not differ significantly between the 2 treatment groups ( Figure 7A). In order to assess whether oxaliplatin treatment alters the phenotype of macrophages in response to a pro-inflammatory stimulus, BMDMs from each mouse were stimulated with TNFα and qRT-PCR was used to probe for a series of marker genes associated with DNA damage, apoptosis and cellular stress. Our data showed no differences between oxaliplatin and vehicle groups, regardless of whether BMDMs had been stimulated with TNFα or not ( Figure 7B). Altogether, our *in vitro* work failed to detect an effect of oxaliplatin on the phenotype of BMDMs. This is line with our flow cytometry findings, where we also failed to detect a change in total CD11b+ cell counts in various tissues.

## Discussion

We set out to test whether alterations in neuro-immune interactions could be observed during the development or maintenance of OIPN in mice. However, despite observing clear signs that oxaliplatin was having a systemic effect, we were unable to detect any substantial oxaliplatin-induced alterations in either pain-like behaviours or the peripheral immune response. Neither acute nor repeated administration of this chemotherapeutic agent majorly influenced the immune profile in neuropathic pain-relevant peripheral tissues including sciatic nerves and lumbar DRG, or in the peripheral inguinal lymph nodes. In keeping with the lack of immune changes, though in contrast to much of the literature, we also failed to observe oxaliplatin-induced pain-like phenotypes in mice receiving multiple doses, namely increased sensitivity to cold and mechanical stimuli. As with all negative data, there are many possible reasons for our failure to detect an effect – including limitations in our own experimental design, but also the suitability and robustness of current animal models of CIPN ( Currie *et al.*, 2019). In the following, we will discuss these issues and highlight possibilities for future investigations.

### No behavioural phenotype observed in mice undergoing repeated oxaliplatin treatment

We failed to detect a behavioural phenotype in mice undergoing repeated cycles of oxaliplatin treatment over a 5-week period, with sensitivity to both mechanical and cold stimuli comparable between drug and vehicle groups. What potential limitations might account for this absence of any effect? Our choice of behavioural assays was based on a multitude of studies which have observed phenotypes consistent with CIPN in rats or mice using very similar von Frey and cold plate paradigms to the ones employed here ( Currie *et al.*, 2019). Furthermore, our data were collected in a blinded fashion by a seasoned experimenter with more than 4 years of experience working with mice. We therefore consider it unlikely that we failed to select appropriate tests or that we carried them out incorrectly.

There are of course subtle differences in how von Frey and cold plate tests are carried out at different laboratories. For example, in the cold plate assay we used “latency to respond” as a read-out, whereas previous studies have relied on “threshold to respond” ( Descoeur *et al.*, 2011; Renn *et al.*, 2011) or the number of responses during a set time period ( Ta *et al.*, 2009). It is therefore possible that very small, specific effects might have been missed in this current study, but still unlikely that a major pain phenotype would have gone unnoticed.

Another limitation is that we cannot be absolutely sure that oxaliplatin reached and damaged sensory neurons. We have circumstantial evidence, since the drug clearly had a significant systemic effect, impeding weight gain. However, we did not employ tissue specific assays. It would therefore have been beneficial to measure platinum concentration in nerve or DRG via atomic absorption spectrometry ( Canta *et al.*, 2011; Marmiroli *et al.*, 2017) or to examine intra-epidermal fibre density (IEFD) as a sign of neuropathy ( Ebenezer *et al.*, 2007; Holland *et al.*, 1997). The latter would have been especially appropriate, considering IEFD has been found to be reduced in the hind-paws of rodents after administration of chemotherapeutic agents, including oxaliplatin ( Boyette-Davis & Dougherty, 2011; Xiao *et al.*, 2012).

Beyond our own study limitations, there are also general issues with how data from animal models of CIPN are being generated and reported. For starters, how exactly OIPN is being induced varies widely from publication to publication. When designing this study, we searched the relevant literature to no avail in hopes of finding a ‘gold standard’ model. Studies typically administer doses of anything between 2–6mg/kg, though a single dose of just 0.5mg/kg has also reportedly proven effective ( Joseph *et al.*, 2008). There is also great variation in injection regimens and routes of administration, e.g. intraperitoneal (i.p.) versus intravenous (i.v.). Importantly, studies investigating the effects of different doses of a given chemotherapeutic agent, including oxaliplatin, have observed that higher doses induce stronger pain-like responses to cold stimuli ( Descoeur *et al.*, 2011; Joseph *et al.*, 2008). Such findings highlight the difficulty in comparing results between laboratories and the need for adoption of standard dosing schedules ( Flatters *et al.*, 2017).

Beyond differences in model induction, one also needs to consider differences in mouse genetic background, since they have been shown to vary in their susceptibility to oxaliplatin as detectable by behavioural, morphological and neurophysiological assessments ( Marmiroli *et al.*, 2017). In fact, of particular interest to this study, the findings of Marmiroli and colleagues suggest that C57BL/6 mice are one of the least susceptible strains, displaying comparatively minimal signs of OIPN, including no cold hyperalgesia. 

Finally, there are issues with how behavioural tests are being conducted. There are clear inter-study inconsistencies, e.g. with the same group reporting use of a 10°C stimulus to assess cold allodynia in two publications ( Ling *et al.*, 2007b; Ling *et al.*, 2007a), but thermal hyperalgesia in another ( Descoeur *et al.*, 2011). Furthermore, temperatures used to assess cold hyperalgesia vary widely - even without performing an in-depth search of the literature, we observed a range from -4.2°C ( Ta *et al.*, 2009) to 10°C ( Descoeur *et al.*, 2011). An additional concern is that investigators continue to use relatively low n numbers in behavioural tests despite high inter- and even intra-animal variability anticipated in most assays. For example, of 1051 mechanical monofilament experiments and 190 cold plate experiments conducted in the CIPN literature, the average sample size was *n*=8 per group (personal communication with Currie et al.). However, with such a sample size it is unlikely that one would be able to detect small effect sizes. For example, using a repeated measures ANOVA with four time points, the minimum effect size that you would be well powered to detect is *f*=0.6. Similarly, using *n*=8 in an unpaired t-test, the minimum effect size one would expect to detect is *d*=1.5. Instead, sample sizes of more than double this (i.e. *n*=16) are required for well-powered detection of effect sizes *d*=1 or larger. ( Extended Figure 4 ( Hore *et al.*, 2021a)). These estimates are based on general parameters and will of course vary depending on the specifics of a particular experiment (such as the precise number of repeated measures). Nevertheless, it is likely that many studies in the field, including our own, will not be designed to detect small differences and will be vulnerable to both false positive and false negative results. The issue of blinding, or lack thereof, is also an important consideration. Although the hope is that every group conducts behavioural tests with certain practises in place, realistically this cannot be monitored. Strikingly, two recent systematic reviews examining 337 ( Currie *et al.*, 2019) and 650 ( Gadgil *et al.*, 2019) CIPN publications revealed that, respectively, only 51.3% and 44% declared that experimenters were blind to treatment group, suggesting that roughly half of all reports in the field run the risk of significant experimenter bias. Lastly, it has been demonstrated that negative data fail to be published in the CIPN field, with many more studies than expected reporting behavioural alterations ( Currie *et al.*, 2019). Indeed, given the statistical framework we all use, even if all studies were powered to detect relevant effect sizes 95% of the time, we would still expect 5% of them to return false negative results. Our study might have been one such instance – and for future meta-analyses, it is crucial to publish and capture each of them.

### Translational potential of current animal models of CIPN

Beyond issues surrounding the execution of animal work in this field, there have been more fundamental concerns as to the suitability of using chemotherapeutics in animals as models for human CIPN. Notably, due to the fact robustness of oxaliplatin-based models is poor across strains and unclear across sex, a recent systematic review did not judge any current OIPN models to be appropriate, preferring instead those involving administration of paclitaxel or cisplatin via a clinically relevant route ( Gadgil *et al.*, 2019).

It is true that numerous discrepancies exist between human and animal phenotypes ( Currie *et al.*, 2019). For example, a key characteristic of CIPN in patients is sensory loss ( Gupta & Bhaskar, 2015; Rice *et al.*, 2018), while animal models almost exclusively report increased sensitivity, particularly to mechanical stimuli ( Hopkins *et al.*, 2016, Lees *et al.*, 2017). Moreover, a recent clinical study of OIPN patients reported that the peak prevalence in mechanical deficits was only reached at 6 months following the end of chemotherapy treatment ( Molassiotis *et al.*, 2019). In contrast, animal studies tend to observe increased sensitivity to mechanical stimuli within one week of oxaliplatin administration ( Gauchan *et al.*, 2009a; Joseph & Levine, 2009; Nassini *et al.*, 2011; Renn *et al.*, 2011). Finally, acute CIPN reportedly affects 68% of patients on average ( Seretny *et al.*, 2014), while animal models using a range of chemotherapeutic agents consistently report behavioural phenotypes in 90–100% of subjects ( Gadgil *et al.*, 2019). Taken together, it therefore appears that type, incidence and onset of symptoms are inconsistent between human CIPN patients and animals in CIPN models. Some of these discrepancies could again be exacerbated by reporting bias, e.g. with studies omitting ‘non-responders’ from their reports without any accompanying explanation or erroneously expecting and therefore detecting a mechanical phenotype due to lack of blinding.

There have been efforts to improve on translatability. For example, tumour bearing animals have been used to investigate the effects of chemotherapy in other contexts ( Hong *et al.*, 2018; Lin *et al.*, 2012). However, to the best of our knowledge, only one such study has attempted to directly study CIPN ( Boyle *et al.*, 2001), despite appropriate models existing for over 20 years ( Boyle *et al.*, 1999). And of course, attempts have been made to better mirror the chronic nature of chemotherapy treatment ( Di Cesare Mannelli *et al.*, 2013; Marmiroli *et al.*, 2017) which typically lasts between 3–6 months and involves 4–8 cycles of treatment (Cancer Research UK, 2018). Accordingly, with 4 cycles over 5 weeks (5% of the lifespan of a mouse), we have tried to adopt such an improved approach here.

### Oxaliplatin only induced minor changes in immune cell numbers in sensory nerves and lymph nodes

With the aforementioned issues in mind, perhaps our flow cytometry experiments were never fit to answer the question of whether neuro-immune interactions are associated with the development and maintenance of OIPN. Still, we can at least conclude that even repeated oxaliplatin treatment did not have a striking effect on leukocyte numbers, with total myeloid and lymphoid cell counts comparable between oxaliplatin and vehicle groups in all tissue types investigated. However, it should be noted that power functions indicate our experiments would likely have only been sensitive enough to detect effect sizes of roughly *d*=1.8 or larger. In other words, we should have had an 80% chance of detecting differences in datasets where 96.4% of the oxaliplatin-treated samples had cell counts higher than the mean of all vehicle samples ( Extended Figure 5 ( Hore *et al.*, 2021a)). Though in actuality we were able to detect effect sizes as low as *d*=1.25 ( Extended Table 2 ( Hore *et al.*, 2021a)), it is likely that we would have missed many other, smaller effects, given the constraints of our current study design.

Despite these limitations, our results are largely in accordance with previous work which found no major alterations in peripheral immune cell composition following repeated oxaliplatin administration. Immunohistochemical analysis revealed that expression of the macrophage/microglial marker IBA1 and the pan-T cell marker CD3, was comparable in the lumbar DRG and sciatic nerves of drug and saline treated mice ( Makker *et al.*, 2017). Prior flow cytometry data on total leukocyte numbers is lacking. While some studies have employed this technique to study immune cells after oxaliplatin administration, they focused on specific immune subpopulations and failed to report on total leukocyte numbers, despite inclusion of an appropriate antibody to do so i.e. CD45 ( Makker *et al.*, 2017; Stojanovska *et al.*, 2018). 

While total myeloid numbers were unchanged, we did observe some subtle effects in specific myeloid sub-populations, though this was tissue dependent. In DRG and sciatic nerve, we noted a reduction in MHCII expressing cells in oxaliplatin treated animals. Although this trend was consistent between models, it only reached significance in the sciatic nerves of mice undergoing repeated administration. In support of this finding, RT-qPCR analysis of whole DRG has revealed downregulation of the MHCII genes H2-Ab1 and H2-Eb1 following 4 weeks of i.v. oxaliplatin administration, an outcome which may suggest oxaliplatin has the ability to selectively damage MHCII+ cells ( Marmiroli *et al.*, 2017). However, the consequences of this reduction in the number of MHCII antigen-presenting cells are unclear. They are known to be essential for the initial activation of CD4+ T cells ( Holling *et al.*, 2004), but we failed to detect any striking changes in lymphocyte numbers in any tissue type investigated ( Extended Figure 2 ( Hore *et al.,* 2021a)). A prior publication described increased CD4+ and CD8+ T cells in the blood after oxaliplatin treatment ( Makker *et al.*, 2017), but like us, failed to detect changes in these populations, or in overall T cell number (CD3+ cells), in the DRG or sciatic nerves. Such results indicate that even if oxaliplatin has the capacity to induce a heightened adaptive immune response, the downstream effects of this are either not large enough to be detected by current study designs or do not manifest in the peripheral nervous tissues relevant for OIPN.

In inguinal lymph nodes, we observed a different picture; following repeated oxaliplatin treatment an increase in almost all myeloid subpopulations was evident, with this reaching significance for the Ly6G+ and MHCII+/Ly6C+ populations. Meanwhile we observed a significant downregulation in MHC-/Ly6C- cells in the oxaliplatin group. Thus, oxaliplatin appeared to cause a reduction in resident myeloid cells but an increase in infiltrating cell types such as monocytes and neutrophils. In contrast, previous work has reported a significant reduction in macrophages and dendritic cells in the mesenteric lymph nodes of oxaliplatin treated mice ( Stojanovska *et al.*, 2018), suggesting that, once more, the effects of oxaliplatin may be tissue-, and in this case, lymph node specific. However, like us, Stojanovska and colleagues did not find any alterations in T cell number. Such results indicate that a myeloid cell shift in the lymph nodes, in either direction, does not appear to have a large effect on adaptive immune cell numbers.

Unlike in the sciatic nerve and DRG, observed trends in total cell number for each population were not consistent between single and repeated administration models in the lymph nodes. Specifically, in the acute model the only difference between groups was noted in the MHCII+ population, where we observed a significant reduction in oxaliplatin-treated mice. These inconsistencies may suggest that, at least within the lymphatic system, immune cell composition is differentially affected by dose, though we would need to repeat these experiments to be certain that they are robust.

## Conclusions

Based on results from this study, we cannot reliably comment on whether neuro-immune interactions are involved in OIPN as we detected no behavioural phenotype and thus no evidence for peripheral neuropathy, even when oxaliplatin was administered in repeated cycles over long periods of time. At least in mice, therefore, we have found this model to be somewhat less robust than other peripheral pain models we have employed in the past, such as partial sciatic nerve ligation (PSNL) ( Denk *et al.*, 2016; Saunders *et al.*, 2018) and intra-plantar administration of complete Freund’s adjuvant ( Lopes *et al.*, 2017; Saunders *et al.*, 2018). While negative data are of course hard to interpret, we nevertheless decided to publish them here to help fight the publication bias widely evident in the current CIPN literature. 

While our behavioural data were inconclusive, our flow cytometry experiments were somewhat easier to interpret. Specifically, in our experiments, oxaliplatin did not appear to have striking effects on peripheral myeloid and lymphoid cell types in lumbar DRG, sciatic nerves or associated lymph nodes. There were only minor changes in myeloid sub-populations, some of which were consistent between our single and repeated administration experiments and with prior literature.

We did not examine the effects on microglia, the resident macrophages of the central nervous system, which play a prominent role in many chronic pain conditions ( Suter, 2016). As it stands, the literature on the effects of oxaliplatin on microglia is conflicting, with a number of immunohistochemical studies reporting increased expression of IBA1 in the dorsal horn of the spinal cord ( Cho *et al.*, 2016; Di Cesare Mannelli *et al.*, 2013; Di Cesare Mannelli *et al.*, 2014), while others report no difference ( Janes *et al.*, 2015; Makker *et al.*, 2017; Zheng *et al.*, 2011).

Finally, our discussions highlighted general limitations with animal models of CIPN. In that context, we think it would be beneficial to streamline protocols between laboratories, increase reporting of methodological details, and make efforts to more closely mimic the types and timing of symptoms experienced by CIPN patients. Furthermore, where feasible, more focus should be put on conducting experiments using CIPN patient derived samples, like blood and surgically resected or post-mortem tissues. Findings from these studies could then be used to test the translational potential of various findings made in animals, moving us one step closer towards understanding the mechanisms underlying CIPN and aiding development of more effective therapeutics.

## Data availability

The data referenced by this article are under copyright with the following copyright statement: Copyright: ï¿½ 2021 Hore ZL et al.

Data associated with the article are available under the terms of the Creative Commons Zero "No rights reserved" data waiver (CC0 1.0 Public domain dedication). 

### Underlying data

Open Science Framework: Probing the peripheral immune response in mouse models of oxaliplatin-induced peripheral neuropathy highlights their limited translatability. https://doi.org/10.17605/OSF.IO/K2SHA ( Hore *et al.*, 2021a).

This project contains the following underlying data:

- Figure 1, Figure 3: Raw mouse weights (XLSX)
- Figure 4 and Figure 5, Extended data Figure 2: Processed flow cytometry data – repeated dosing (XLSX)
- Figure 2, Extended data Figures 2 and 3: Processed flow cytometry data – single dose (XLSX)
- Figure 6: Raw behavioural data (XLSX)
- Figure 7A: Raw BMDM counts (XLSX)
- Figure 7B: Raw BMDM RT-qPCR data (XLSX)

Open Science Framework: Repeated dosing flow cytometry - FCS files and workspace. https://doi.org/10.17605/OSF.IO/P2Y5Z ( Hore *et al.*, 2021b).

This project contains the following underlying data:

- Figure 4 and Figure 5 and Extended data Figure 2: 36 FCS files generated in flow cytometry experiments on nerve, DRG and lymph node tissues (FCS)
- Figure 4 and Figure 5 and Extended data Figure 2: 8 FCS files of fluorescent minus one ‘FMO’ controls used for gating purposes in flow cytometry experiments (FCS)
- Figure 4 and Figure 5 and Extended data Figure 2: Total numbers gating – repeated dosing (WSP). This is a FlowJo workspace where all samples were gated and total cell numbers were generated.

Open Science Framework: Single dose flow cytometry - FCS files and workspace. https://doi.org/10.17605/OSF.IO/QFDRN ( Hore *et al.*, 2021c).

This project contains the following underlying data:

- Figure 2, Extended data Figures 2 and 3: 36 FCS files generated in flow cytometry experiments on nerve, DRG and lymph node tissues (FCS). Note that vehicle animals are labelled as ‘PBS’.
- Figure 2, Extended data Figures 2 and 3: 8 FCS files of fluorescent minus one ‘FMO’ controls used for gating purposes in flow cytometry experiments (FCS)
- Figure 2, Extended data Figures 2 and 3: Total numbers gating – single dose (WSP). This is a FlowJo workspace where all samples were gated and total cell numbers were generated.

### Extended data

Open Science Framework: Probing the peripheral immune response in mouse models of oxaliplatin-induced peripheral neuropathy highlights their limited translatability. https://doi.org/10.17605/OSF.IO/K2SHA ( Hore *et al.*, 2021a).

This project contains the following extended data:

- **Extended Figure 1 (PDF). Representative gating strategies employed for flow cytometry experiments.** (A) DRG (sample displayed: vehicle, chronic model, second processing day), (B) Sciatic nerves (sample displayed: vehicle, chronic model, second processing day), (C) Inguinal lymph nodes (sample displayed: oxaliplatin, chronic model, second processing day). For all tissue types, gating was as follows: CD45+ vs FSC-A for CD45+ events; SSC-A vs FSC-A for CD45+ cells i.e. leukocytes; FSC-W vs FSC-A for single cells; Live/Dead- vs FSC-A for live cells; FSC-A v CD11b for myeloid cells (CD11b+) OR Ly6G vs CD11b for neutrophils (CD11b+, Ly6G+) and lymphoid cells (CD11b-, Ly6G-). From the myeloid cell population with neutrophils excluded (CD11b+, Ly6G-): MHCII vs Ly6C for MHCII antigen presenting cells (MHCII+, Ly6C-), resident macrophages (MHCII-, Ly6C-), infiltrating monocytes (MHCII, Ly6C+) and a double positive population which we propose to be infiltrating monocytes differentiating into resident populations (MHCII+, Ly6C+). From the lymphoid population (CD11b-, Ly6G-): βTCR vs γδTCR for αβ+ T cells (βTCR+, γδTCR-) and γδ+ T cells (γδTCR+, βTCR-).
- **Extended Figure 2 (PDF). Total numbers of lymphoid and lymphoid subpopulation cells did not differ between vehicle and oxaliplatin treated mice in any tissue type either after acute or repeated oxaliplatin administration.**Analysis of total number of lymphoid (CD45+/CD11b-/Ly6G-), αβ+ T cells (CD45+/CD11b-/Ly6G-/βTCR+, γδTCR-) and γδ+ T Cells (CD45+/CD11b-/Ly6G-/βTCR-, γδTCR+) from flow cytometry of (A) Lumbar 3–5 dorsal root ganglia: (i) acute administration, (ii) repeated administration. (B) Sciatic nerves: (i) acute administration, (ii) repeated administration. (C) Inguinal lymph nodes: (i) acute administration, (ii) repeated administration. Data displayed as individual animals ±SEM, ( *n*=5–6). Unpaired t-test or Mann-Whitney U test, depending on whether data was normally distributed (Shapiro-Wilk test).
- **Extended Figure 3 (PDF). Total myeloid cell numbers did not differ between vehicle and oxaliplatin treated mice in any tissue type 4 days following a single dose of oxaliplatin (6mg/kg).** Flow cytometry revealed no significant difference in total number of myeloid cells (CD45+/CD11b+) for any tissue type. Similarly, no significant dysregulation was observed in any myeloid lineage subpopulation investigated, with the exception of MHCII antigen presenting cells (CD45+/CD11b+/Ly6G-/MHCII+, Ly6C-), which were downregulated the inguinal lymph nodes of oxaliplatin treated mice. Analysis of flow cytometry results from (A) Lumbar 3–5 dorsal root ganglia. (B) Sciatic nerves. (C) Inguinal lymph nodes. Data displayed as individual animals ±SEM, ( *n*=5–6). Unpaired t-test or Mann-Whitney U test, depending on whether data was normally distributed (Shapiro-Wilk test), * p<0.05, ** p<0.01.
- **Extended Figure 4 (PDF).** Plotted power functions give an idea of the smallest effect size the average ( *n*=8) von Frey and cold plate experiments conducted in the CIPN literature would have had an 80% chance of detecting (any x-axis number at or to the right of the dotted black vertical lines), (A) based on the use of an unpaired t-test, (B) based on the use of a repeated measures ANOVA. As this was only the average sample size, we have also included a range above and below this ( *n*=6 - *n*=16), the largest of which ( *n*=16) would be powered to detect effect sizes equal to, or larger than *d*=1 and *f*=0.4. Note that for both tests, the default parameters of G*Power were used to give a general idea. Of course, in actuality these tests would be tailored to the specifics of a particular experiment.
- **Extended Figure 5 (PDF).** Sensitivity curves give an idea of the smallest effect sizes we would have been likely to detect in our flow cytometry experiments with n = 6 (80% chance of detection for any x-axis values at or to the right of the dotted black vertical line. These power calculations are for a parametric unpaired t-test (A) and a non-parametric Mann-Whitney test (B), to mirror those used in our analyses.
- **Extended Table 1 (DOC). **List of animals excluded from experimental analysis.
- **Extended Table 2 (DOC).** Effect sizes of all comparisons which resulted in statistically significant differences. Selected non-significant examples from the myeloid cell population (CD45+/CD11b+) are also given for comparative purposes. Extended Figure 5 provides the minimum effect sizes for which these statistical tests would have been well-powered (i.e. 80%), given our total sample size of 12.

Data are available under the terms of the Creative Commons Zero "No rights reserved" data waiver (CC0 1.0 Public domain dedication).

### Reporting guidelines

Open Science Framework: The ARRIVE guidelines 2.0: author checklist for ‘Probing the peripheral immune response in mouse models of oxaliplatin-induced peripheral neuropathy highlights their limited translatability’. https://doi.org/10.17605/OSF.IO/K2SHA ( Hore *et al.*, 2021a)

Data are available under the terms of the Creative Commons Zero "No rights reserved" data waiver (CC0 1.0 Public domain dedication).
