# Supplementary material for: Probing the peripheral immune response in mouse models of oxaliplatin-induced peripheral neuropathy highlights their limited translatability
Source: Wellcome Open Res. 2021 Jun 11;6:68. Originally published 2021 Mar 29. [Version 2] doi: 10.12688/wellcomeopenres.16635.2 (PMC8243229; doi:10.12688/wellcomeopenres.16635.2)
Supplement: Peer review history

# Peer review: Reviewer response for version 2

**DOI:** 10.21956/wellcomeopenres.18722.r44396

**Published:** 2021-06-29

**Authors:** Emily Sena, Gillian Currie

**Affiliations:** 1 Stroke Association Kirby Laing Foundation Senior Non-Clinical Lecturer, Centre for Clinical Brain Sciences, University of Edinburgh, Edinburgh, UK; 2 Centre for Clinical Brain Sciences, University of Edinburgh, Edinburgh, UK

The authors’ revision have improved the manuscript and we are delighted that they found our comments to be of use.

 We have two minor comments for the author’s to consider but are very happy with this new version of the manuscript.

**2. Randomisation**

 Thanks for the clarification; however, we would encourage you to remove “computer-based” as arbitrary assignment is not a form of randomisation.

**6. Power functions**

 Thanks, this is much clearer and we better understand your approach. We must admit that we still think the description of the power functions and figure 4 should be included in the results sections.

Is the work clearly and accurately presented and does it cite the current literature?

Yes

If applicable, is the statistical analysis and its interpretation appropriate?

Partly

Are all the source data underlying the results available to ensure full reproducibility?

Yes

Is the study design appropriate and is the work technically sound?

Partly

Are the conclusions drawn adequately supported by the results?

Yes

Are sufficient details of methods and analysis provided to allow replication by others?

Yes

Reviewer Expertise:

experimental validity, preclinical bio-curation, meta-research

We confirm that we have read this submission and believe that we have an appropriate level of expertise to confirm that it is of an acceptable scientific standard.

# Peer review: Reviewer response for version 2

**DOI:** 10.21956/wellcomeopenres.18722.r44395

**Published:** 2021-06-15

**Authors:** Margarita Calvo

**Affiliations:** 1 Department of Physiology, Faculty of Biological Sciences, Pontificia Universidad Católica de Chile, Santiago, Chile; 2 MINUSPAIN (milenium nucleus for the study of pain) Iniciativa milenio, Santiago, Chile

I think the manuscript has greatly improved and now is ready for indexing.

Is the work clearly and accurately presented and does it cite the current literature?

Partly

If applicable, is the statistical analysis and its interpretation appropriate?

Yes

Are all the source data underlying the results available to ensure full reproducibility?

Yes

Is the study design appropriate and is the work technically sound?

Yes

Are the conclusions drawn adequately supported by the results?

Yes

Are sufficient details of methods and analysis provided to allow replication by others?

Yes

Reviewer Expertise:

Neurobiology of pain

I confirm that I have read this submission and believe that I have an appropriate level of expertise to confirm that it is of an acceptable scientific standard.

# Peer review: Reviewer response for version 1

**DOI:** 10.21956/wellcomeopenres.18340.r43304

**Published:** 2021-04-29

**Authors:** Emily Sena, Gillian Currie

**Affiliations:** 1 Stroke Association Kirby Laing Foundation Senior Non-Clinical Lecturer, Centre for Clinical Brain Sciences, University of Edinburgh, Edinburgh, UK; 2 Centre for Clinical Brain Sciences, University of Edinburgh, Edinburgh, UK

Hore and colleagues describe their investigation of oxaliplatin-induced peripheral neuropathy following single or repeated oxaliplatin administration. In the repeated dosing model they assessed for mechanical and thermal pain-related behaviours. They also investigated whether there were changes to peripheral immune cells in these models. This study did not identify changes in leukocyte, lymphocyte or myeloid cell numbers in DRG, sciatic nerves or inguinal lymph nodes. Small changes in myeloid cells were identified. Importantly, the authors highlight that their results are not consistent with what has been reported in the field.

 We applaud you, this is an excellent example of transparent reporting, and it is encouraging to read such study, especially as the findings do not agree with the published literature. This is a really important step to tackle publication bias (as the authors note). Further, you have shared the raw data from your study (via Open Science Framework).

 You consider in detail the impact of study design limitations (e.g. small sample size) and report your results with this in mind. This is an important study for the field highlighting the need to appropriately power experiments due to inter and intra animal variability of behavioural assays. You provide excellent detail on the measures used to ameliorate harm to animals used in the study and you give details of attrition (not common across the CIPN literature).

 Some thoughts for you to consider:

1. Where you state the study is reported in line with the ARRIVE guidelines, we would encourage you to be specific that the citation is to their checklist.
2. Randomisation - you use the term “arbitrarily assigned”? It is unclear whether this is true randomisation?
3. You do not adjust your p-value for multiple testing, we would encourage this ( e.g. figure 5). Although given that outcomes were not sufficiently powered or no primary outcome is stated, inferential statistics are probably not appropriate.
4. Related to the above point, you based your sample size on previous studies. If previous studies were underpowered (which is likely) this leads to a persistent problem that is likely a domain-wide issue. However, you do note the limitation of using a small sample size in your discussion.
5. Where live and re-scored latencies differed, the re-scored time was taken but is there no possibility this value was erroneous?  
6. For the power functions, it is unclear which data were used. You describe “published behavioural tests”, where do these come from? These findings are first described in the discussion, they should probably be included in the results sections.
7. You have essentially used “Weight” as an outcome, we suggest including this in the outcome measures list.
8. Where you state “data not shown”, is it possible to add this to the OSF project for completeness?
9. We appreciate that “negative data” are how such findings are often described but we think this is unnecessarily pejorative. Given the working hypothesis (no formal hypothesis is stated), you have accepted the null hypothesis. We also assert that the limitations in experimental design you describe could lead to either accepting or rejecting the null hypothesis, it is just that in cases like this we try to find reasons for the findings observed. We would encourage you to re-frame this as all studies, irrespective of their findings, should be taking a similar approach as you have done here.
10. You suggest that we should having a standard dosing schedule for oxaliplatin. We can imagine that different dosing schedules may be required in certain circumstances and it may be more useful would be to better understanding of the effect of different dosing schedules?

Is the work clearly and accurately presented and does it cite the current literature?

Yes

If applicable, is the statistical analysis and its interpretation appropriate?

Partly

Are all the source data underlying the results available to ensure full reproducibility?

Yes

Is the study design appropriate and is the work technically sound?

Partly

Are the conclusions drawn adequately supported by the results?

Yes

Are sufficient details of methods and analysis provided to allow replication by others?

Yes

Reviewer Expertise:

experimental validity, preclinical bio-curation, meta-research

We confirm that we have read this submission and believe that we have an appropriate level of expertise to confirm that it is of an acceptable scientific standard, however we have significant reservations, as outlined above.

# Response

**Published:** 2021-06-02

**Authors:** Zoe Lee Hore

Thank you for the supportive feedback regarding our manuscript. We greatly appreciate you taking the time to bring to our attention a number of important points and hope that we have addressed your concerns with our revised manuscript or with our responses below.  

**1. Where you state the study is reported in line with the ARRIVE guidelines, we would encourage you to be specific that the citation is to their checklist.**

 Apologies for this important oversight. In the ‘Ethical Considerations’ section we have now cited the Percie du Sert *et al.* (2020) paper these guidelines derive from. Additionally, the link “ Hore et al 2021a” provided in our MS takes you to OSF where we have uploaded a PDF of the Full ARRIVE 2.0 Checklist where we note the section of our manuscript in which each recommendation is addressed (Files > Archive of OSF storage > ARRIVE guidelines 2.0 - Author Checklist).

**2. Randomisation - you use the term “arbitrarily assigned”? It is unclear whether this is true randomisation?**

 As we did not employ computer-based randomisation, we cannot term our approach true randomisation and have thus deemed it “arbitrary assignment”. We have now explained more thoroughly how animals were assigned in the methods section to clarify what we mean by the term “arbitrary”.

**3. You do not adjust your p-value for multiple testing, we would encourage this (e.g. figure 5). Although given that outcomes were not sufficiently powered or no primary outcome is stated, inferential statistics are probably not appropriate.**

 We completely agree. We followed the convention of most pre-clinical work by using inferential statistics on samples sizes that are arguably too small to be meaningful (given the expected effect sizes) and using unadjusted p-values when adjusted ones would have been more appropriate. To highlight this potential issue, we have now added a paragraph to our discussion – just before the "Conclusion" section.

**4. Related to the above point, you based your sample size on previous studies. If previous studies were underpowered (which is likely) this leads to a persistent problem that is likely a domain-wide issue. However, you do note the limitation of using a small sample size in your discussion.**

 This is very true and indeed, in hindsight, we should not have based sample sizes on previous publications, but instead on smallest effect size of interest, as highlighted in Albers and Lakens (2018). We have now added this to our discussion in the section on behavioural phenotypes.

**5. Where live and re-scored latencies differed, the re-scored time was taken but is there no possibility this value was erroneous?**

 This is a possibility; however, we would argue that scoring from the video will always provide a more accurate value than live scoring. During re-scoring, videos could be paused and re-watched to ensure a true response was recorded. Indeed, in many cases, where responses were subtle, videos were re-watched multiple times to ensure accuracy of the latency recorded. Additionally, in some instances a live score was not recorded as it was difficult to observe the animal without being too intrusive or no clear responses were made during the 90 seconds. In these instances, scoring solely from the video recording was the only option. Therefore, in order to be consistent, we decided it would be most appropriate to take the re-scored values for all instances. 

**6. For the power functions, it is unclear which data were used. You describe “published behavioural tests”, where do these come from? These findings are first described in the discussion, they should probably be included in the results sections.**

 Our power functions are simple sensitivity analyses. We are not using any particular data/studies, instead we are determining the mere mathematics of which effect size a given statistical test is powered to detect given different samples sizes, ranging from what is a below average n number for a behavioural study in the field (e.g. n = 6) to what is an above average number for a behavioural study (e.g. n = 16). We know from your work that n = 8  is average in the field.

 Given the theoretical nature of these sensitivity analyses, we therefore did not really consider them results as such. We have now tried to clarify this in our “statistical analysis” section by separating the paragraph on actual observed effect sizes based on our data from that on the theoretical sensitivity analyses. We also dropped the word “published” – confusing in this context.

**7. You have essentially used “Weight” as an outcome, we suggest including this in the outcome measures list.**

 Apologies for this oversight, we have now added this to our outcome measures list.

**8. Where you state “data not shown”, is it possible to add this to the OSF project for completeness?**

 Of course - we have now uploaded Extended Table 3 to OSF, where data comparing overall leukocyte numbers to behavioural phenotype for each animal is provided.

**9. We appreciate that “negative data” are how such findings are often described but we think this is unnecessarily pejorative. Given the working hypothesis (no formal hypothesis is stated), you have accepted the null hypothesis. We also assert that the limitations in experimental design you describe could lead to either accepting or rejecting the null hypothesis, it is just that in cases like this we try to find reasons for the findings observed. We would encourage you to re-frame this as all studies, irrespective of their findings, should be taking a similar approach as you have done here.**

 All uses of the term “negative data” have now been altered to avoid using what we agree to be a pejorative term.

**10. You suggest that we should having a standard dosing schedule for oxaliplatin. We can imagine that different dosing schedules may be required in certain circumstances and it may be more useful would be to better understanding of the effect of different dosing schedules?**

 This is a very good point, thank you for bringing it to our attention. Though we do comment on the fact that different dosing regimens all appear to result in similar behavioural phenotypes (please see our newly added Extended Table 4), we also note that higher doses reportedly induce stronger pain-like responses. Thus, it is clear that more investigation to compare different dosing schedules is required in order for researchers to be able to decide upon the most appropriate regimen for their particular study. Accordingly, we have now worked this into our discussion under the section “No behavioural phenotype observed in mice undergoing repeated oxaliplatin treatment”.

# Peer review: Reviewer response for version 1

**DOI:** 10.21956/wellcomeopenres.18340.r43302

**Published:** 2021-04-12

**Authors:** Margarita Calvo

**Affiliations:** 1 Department of Physiology, Faculty of Biological Sciences, Pontificia Universidad Católica de Chile, Santiago, Chile; 2 MINUSPAIN (milenium nucleus for the study of pain) Iniciativa milenio, Santiago, Chile

The article "Probing the peripheral immune response in mouse models of oxaliplatin-induced peripheral neuropathy highlights their limited translatability" is a very clear report of a negative study on Oxaliplatin induced neuropathy in a mouse model. I agree with the authors that publication of data like this is very important for the field as it help to improve models of neuropathic pain and because it reduces publication bias.

 The methods and the analysis are very detailed, allowing for reproducibility of the data, which is very welcome and not very common. 

 My only suggestion would be to include in the discussion data regarding OIPN in rats, and how this compares to mouse models. It is important to try to understand which model of OIPN is best fitted to reproduce the clinical problem, and therefore, a comparative with data published in other rodents might be very useful. I would also suggest to make a detailed comparison of the protocols used to administer oxaliplatin in the different models, as this varies across laboratories and might be key determinants of development of neuropathy.

 I agree that nothing can be said in terms of immune system recruitment in OIPN with this model, as it does not reproduce the neuropathy seen in patients. 

 Maybe it is worth in this article to give suggestions to other researchers on what are the best models for studying OIPN in the opinion of the authors: species to use, protocol and dose of oxaliplatin administration, and outcomes to measure. This may help other researchers to use adequate models, and to avoid models which have proven not to mimic the clinical condition, in order to understand the mechanism behind OIPN.

Is the work clearly and accurately presented and does it cite the current literature?

Partly

If applicable, is the statistical analysis and its interpretation appropriate?

Yes

Are all the source data underlying the results available to ensure full reproducibility?

Yes

Is the study design appropriate and is the work technically sound?

Yes

Are the conclusions drawn adequately supported by the results?

Yes

Are sufficient details of methods and analysis provided to allow replication by others?

Yes

Reviewer Expertise:

Neurobiology of pain

I confirm that I have read this submission and believe that I have an appropriate level of expertise to confirm that it is of an acceptable scientific standard, however I have significant reservations, as outlined above.

# Response

**Published:** 2021-06-02

**Authors:** Zoe Lee Hore

Thank you for the positive feedback and for bringing to our attention some additions that could be made to improve the utility of our manuscript to the scientific community working in this area.

 To address the concerns you have raised, we have made the following changes to the manuscript:

 Firstly, we have now added Extended Table 4 to our Extended Data on OSF, which compares all of the models used in papers we have referenced in our manuscript. This table highlights differences in administration route, dose and the resulting phenotypes observed in both single and repeated dose models in mice and rats. In addition, we have referenced two review papers which have compared a number of oxaliplatin-based models used across the two species.

 In relation to these comparisons, we have now noted in our discussion that models used between rodent species all appear to result in similar phenotypes, whether the drug is administered acutely or in repeated cycles. We also make the point that while no models in the current OIPN literature appear to mimic the clinical problem exactly, repeated dosing at least mimics clinical administration schedules. However, as it stands, the literature is clearly “missing” data on when model induction failed. It is therefore difficult to gauge the proportional “success” of each type of dosing regimen. As such, we unfortunately do not feel as though we can currently make a very informed choice on the most appropriate model for studying OIPN. We state this clearly in the text.
